# Loss of Paired Weapons Leads to Larger Testes and a Lighter Load for Dispersal

**DOI:** 10.1002/ece3.71724

**Published:** 2025-07-06

**Authors:** James C. Boothroyd, Steve M. Smit, Niko S. Zlotnik, Christine W. Miller

**Affiliations:** ^1^ Entomology & Nematology Department University of Florida Gainesville Florida USA; ^2^ Kenai Peninsula College University of Alaska Anchorage Soldotna Alaska USA; ^3^ Department of Zoology University of Cambridge Cambridge UK

## Abstract

Reproduction is often costly for males, as it may require the growth of structural traits that aid in dispersal to find females, competition over mating opportunities, and ejaculate production. The growth of such traits can be energetically demanding, and these demands often arise concurrently during development. As such, these traits may be especially prone to resource allocation trade‐offs. Yet, such traits are rarely studied in tandem. We designed a study to improve understanding of investment dynamics in flight muscle, a dispersal trait; a sexually selected weapon used in mate competition; and testes used for sperm production. We used the leaf‐footed cactus bug, *Narnia femorata* (Hemiptera: Coreidae), a species where males use their hindleg as weapons to compete for matings. Males can naturally drop their limbs, and when hindlegs are lost during development, adult males do not grow a weapon. Existing studies have revealed that testes growth increases when investment in weapons ceases. Yet, this work only examined responses to the loss of a single hindleg and limited the scope of traits to testes. Here, we examined weapon loss at two levels and investigated a third trait: dispersal. We found that testes size increased stepwise with limb loss; the loss of one hindleg weapon increased testes mass by around 9%, and two legs increased it by 20%. This intriguing pattern suggests a direct, quantity‐specific trade‐off in tissue development across traits. We also detected only a limited increase in dispersal investment when males did not grow weapons. Yet, dispersal may still be enhanced for those that drop hind legs; those without the substantial weight of hind limbs may have the potential to disperse farther.

## Graphical abstract

We addressed a weapons testes trade‐off using natural limb loss as an experimental manipulation of weaponized legs in the leaf‐footed cactus bug. We found that individuals missing weapons invested in their testes proportional to the number of weaponized legs they lost. We also investigated a third trait, dispersal, and found that individuals missing weapons did not alter their level of investment in flight muscle mass but should be capable of flying further due to their lack of bulky weapons.

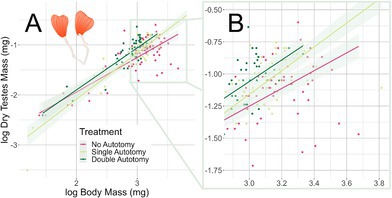

## 1 Introduction

Males face many challenges in achieving reproduction. These challenges can include dispersing to find potential mates, competing against rivals, and producing sufficient sperm to fertilize a female's eggs. In insects and other flying animals, dispersing successfully typically requires investment in wings and flight musculature (Marden 1987). After locating females, many males engage in physical contests with other males to even have a chance at a potential mating (Emlen and Oring 1977; Shuster and Wade 2003). Enlarged sexually selected weapons have commonly evolved for such contests, like the antlers of elk and the horns of many dung beetles (Emlen 2008). Further, the ejaculates of males may face competition in those cases where females mate with multiple partners, potentially reducing the share of eggs each male is able to fertilize (Parker 1970; Simmons 2001). Males in species with intense sperm competition often grow large testes and produce more or more competitive sperm (Pitnick 1996; Vahed et al. 2011; Vahed and Parker 2012; Vrech et al. 2014; Lüpold and Fitzpatrick 2015; Parker 2016; Lüpold et al. 2020; Degueldre and Aron 2023). The process of building and maintaining the physical structures responsible for dispersal (Marden 1989; Zera and Denno 1997; Zera and Harshman 2001), ejaculates (Wedell et al. 2002; Simmons et al. 2017; Greenway et al. 2020) and fighting (Allen and Levinton 2007; Somjee, Woods, et al. 2018; O'Brien et al. 2019; but see McCullough and Tobalske 2013; Toigo et al. 2013) is often energetically expensive.

Energetically demanding structures, such as the ones described, are expected to compete for internal resources, and this process can lead to resource allocation trade‐offs (Perrin and Sibly 1993; Parker et al. 2013). Such trade‐offs may be especially pronounced when multiple traits are grown at the same developmental stage (Emlen 2001; Roff and Fairbairn 2007; Miller et al. 2019; Garland et al. 2022). For example, investment in flight muscles can be negatively associated with concomitant gamete production in females (Mole and Zera 1993; Tanaka 1993; Zera et al. 1998; Cheng et al. 2016) and male insects (Heinze and Hölldobler 1993; Langellotto et al. 2000; Saglam et al. 2008). Research on trade‐offs is important as it can provide insights on investment priorities, flexibility, and constraints during development.

This study had two major objectives. Our first objective was to better understand the resource allocation dynamics affecting flight muscle, weapons, and testes. We selected these traits because they each play important roles in male reproduction, they have high investment costs for males, and they are rarely measured in the same study. In many species, flight muscle, weapons, and testes are grown at approximately the same developmental window, so resource allocation trade‐offs might be expected. Our second objective was to better understand the energetic relationships between traits, particularly precopulatory weapons and testes, using an established trade‐off (Joseph et al. 2018; Somjee, Miller, et al. 2018; Miller et al. 2019, 2021; Greenway et al. 2023).

Males in many leaf‐footed bugs establish territories on host plants using their enlarged hind legs (Mitchell 1980; Fujisaki 1981; Eberhard 1998; Okada et al. 2011; Procter et al. 2012). Males remain on territories for several days, mating with females and fighting with males before eventually dispersing to another territory to repeat the process (Fujisaki 1981; Miller 2007). Leaf‐footed bugs are typically not long‐distance fliers (Mann 1969), but instead piece together short flights during the process of dispersal. They have been recorded dispersing multiple miles (Mann 1969; Miller 2007), and in one case, 92% of marked adults were found on different host plants than where they were first sighted (Miller 2007). The leaf‐footed cactus bug, *Narnia femorata* Stål (Hemiptera: Coreidae), often lives on highly patchy prickly pear cacti (*Opuntia*. Spp.), and must navigate spaces between host plants. Thus, flight musculature represents an important point of investment, especially for males.

Insects in the leaf‐footed bug family commonly autotomize, or willingly drop their limbs, after which these legs do not regrow (Emberts et al. 2017). In natural populations of *N. femorata*, around 13% of individuals are missing at least one leg (Emberts et al. 2016). Male *N. femorata* missing weaponized hind limbs are at a disadvantage in competitions, rarely winning fights with intact males (Emberts et al. 2018). Previous work in this species has shown that males experience resource‐allocation trade‐offs between their weapons and testes. Those prevented from growing a weapon via autotomy grow bigger testes (Joseph et al. 2018). Further, the increase in mass seen in the testes of autotomized males is due to an increase in sperm production (Cavender et al. 2021), which translates to a reproductive advantage in non‐competitive scenarios (Cirino et al. 2021). Females store sperm (Allen et al. 2018) and mate multiply (Emberts et al. 2018; Greenway et al. 2021), suggesting sperm competition is at play. Autotomy status does not appear to impact female preference (Greenway et al. 2023); greater sperm production could yield a competitive advantage.

Here, we examined resource allocation trade‐offs among flight muscle, weapons, and testes using autotomy as an experimental tool. Our work follows others that have used experimental manipulation to reveal trade‐offs (Sinervo and DeNardo 1996; Soler et al. 2003). Such research has shown that, for example, the inhibition of weapon development can result in larger testes (Simmons et al. 2017; Joseph et al. 2018; Somjee, Miller, et al. 2018), and cauterizing genital disks can result in larger weapons (Moczek and Nijhout 2004). In *N. femorata* , investment in weapons can be manipulated with ease without causing a detectable increase in mortality (Joseph et al. 2018; Miller et al. 2019). We provide the first study to manipulate investment in precopulatory weapons at multiple levels to examine the investment outcome for other traits.

Here, we induced the loss of one or both weaponized hind legs in *N. femorata* at the penultimate juvenile stage. Although only around 2% of individuals are naturally missing two legs (Emberts et al. 2016), the ability to inhibit investment in weapons at two levels enables a more granular examination of the nature of resource allocation trade‐offs. We hypothesized that males that are prevented from a developmental investment in weapons should divert resources to other expensive traits developing at the same time—flight musculature and testes. Further, we predicted a larger increase in investment in these other traits when males were prevented from developing both hind‐leg weapons. A trade‐off between weapons and testes has already been documented in this species, and here, we tested if the increase in testes mass would be doubled when males were prevented from growing both hind limb weapons versus just one.

## 2 Materials and Methods

### 2.1 Insect Rearing

The insects used in this study descended from 62 pairs of wild‐caught and laboratory‐reared parents that originated from a native population of *Narnia femorata* in Phoenix, Arizona and an introduced population in Florida. Each pair was composed of a Florida and Arizona individual. This inter‐population crossing was done to expand genetic diversity in the population, as the Florida population has limited genetic variance (Sasson et al. 2016). Juveniles were kept in groups with up to seven siblings. When a juvenile reached its penultimate instar, it was removed and assigned at random to one of three treatments: no autotomy (intact; NA), where individuals kept both hind legs; single autotomy (SA), where individuals had only their left hind leg autotomized; or double leg autotomy (DA), where both hind legs were autotomized. Autotomy was induced by gently grasping the hind leg at the proximal end of the hind femur with reverse‐action forceps, until the insect released its leg. If NA was required, the left leg was brushed several times as a control. Hemipteran insects undergo rapid growth during their penultimate instar, investing heavily in adult structures like reproductive organs (Wick 1954; Amukamara et al. 2020). Further, juveniles begin investing in their hind legs late in juvenile development, making this an ideal stage at which to manipulate investment (Greenway et al. 2023). After a treatment was applied, individuals were transferred to their own cup to finish maturation individually, to ensure they remained unmated. We did not observe any difference in activity or feeding behavior in doubly autotomized individuals. Males were frozen 14 days after eclosing into adulthood to ensure they were all sexually mature. Insects were kept in lidded, clear plastic enclosures (14.5 cm high, 11 cm in diameter), with a single eastern prickly pear (*Opuntia* spp.) cladode and attached fruit planted in soil. We kept pairs in a 14:10 (light: dark) photoperiod with temperatures ranging from 33°C in the daytime to 25°C at night, and an average humidity of 65%. Insect collection and rearing occurred in the summer and fall of 2020, with data collection for this study the following year. All experimentation took place on the University of Florida campus in Gainesville, Florida.

### 2.2 Dissection

**FIGURE 1:**
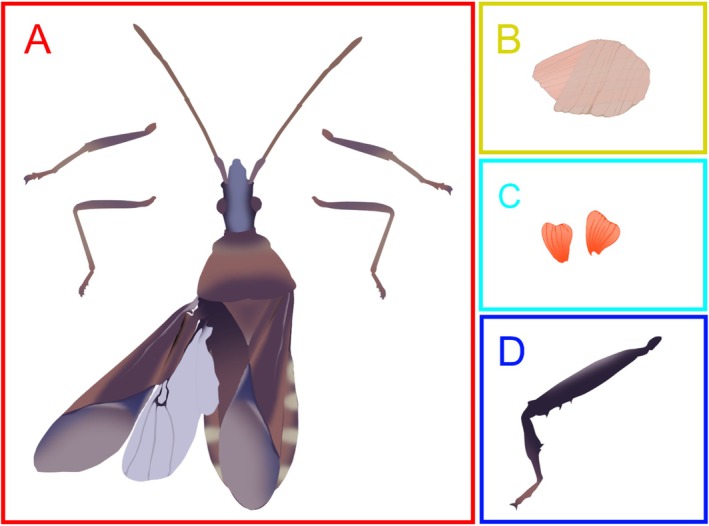
Illustrations of the body parts that were used in this study. After dissection, the wings, foreleg and midleg pairs, and the bodies of insects were dried and weighed together (A). The dissected flight muscles (B), both testes (C), and remaining hind legs (D) were each weighed separately. In Analysis 1, body mass was only the mass of A. In Analysis 2, our covariate body mass was composed of the mass of A plus B. In Analysis 3, the covariate body size was composed of the mass of A plus C. In Analysis 4, the covariate total lift mass was composed of the mass of A plus C and D. In Analysis 4, body mass included the mass of A.

Frozen insects were dissected in two rounds. First, the testes were removed from the abdomen, hind limbs were removed, and the thorax and head were separated from the rest of the body. All body parts were stored in 70% EtOH at 4°C. After at least 24 h, when flight muscles had been suitably fixed, dissections on the thorax continued, freeing the flight muscles from the dorsal nota before storing them in the same manner. All tissues (testes, hind limbs, flight muscles, and remaining body parts) were dried for at least 48 h at 60°C and then weighed using a microbalance (Mettler Toledo XP6: Columbus, OH, USA), to the nearest microgram. We used body mass as our primary proxy for body size (Figure 1) and the width of the insect's pronotum as a secondary proxy (Gillespie et al. 2014) (Text S1).

### 2.3 Statistical Analyses

For Analysis 1, we used the lavaan package to do a path analysis (Version: 0.6.19; Rosseel 2012). We constructed linear mixed models (LMMs) for each of Analyses 2, 3, and 4 (Package: lme4; Version: 1.1.35.1) (Bates et al. 2015). Testes and flight muscle mass should increase with body size, and thus body mass was used as a covariate in our analyses. We incorporated parentage as a random effect in Analyses 2, 3, and 4, and we used a Wald Chi square test to detect statistical significance (Package: rstatix; Version: 0.7.2) (Kassambara 2023). All statistical analyses were performed using R version 4.3.2 (2023‐10‐31 ucrt) (R Core Team 2021). To improve normality, heteroskedasticity, and linearity, all variables were log transformed prior to analysis. Log transformation also makes values scale‐independent, enabling easier comparison of scaling relationships (Huxley 1924; Shingleton and Frankino 2013). Although quartiles were used for visual assessment of our data, we did not exclude any individuals from our formal analyses based on trait value.

#### 2.3.1 Analysis 1

**FIGURE 2:**
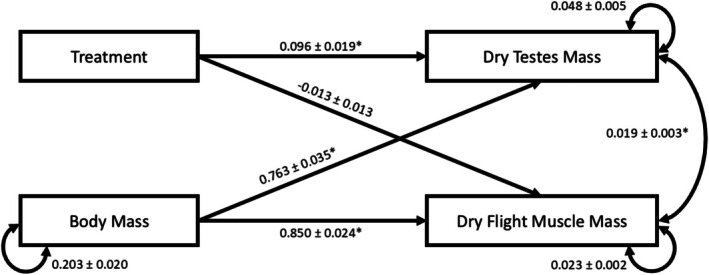
The results of our path analysis showing a small but significant positive covariance between testes mass and flight muscle mass, when controlling for treatment and body mass. Single‐headed arrows indicate causal relationships, and double‐headed arrows indicate correlations between traits, or variances of a single trait. Correlation coefficients are shown along lines, with standard errors. Asterisks indicate statistical significance.

We first examined the trade‐off holistically by performing a path analysis, including both testes mass and flight muscle mass as response variables. We hypothesized that both body size and autotomy status (NA, SA, or DA) would share a positive, linear relationship with both testes mass and flight muscle mass (Figure 2; single‐headed arrows). To avoid part‐whole correlation, only the mass of the body was used, not including flight muscle, testes, or remaining weapon mass (Figure 1A) (Tomkins and Simmons 2002). The covariance between flight muscle mass and testes mass was included to determine the correlative relationship between these two energetically costly traits; however, as neither variable was directly manipulated, we did not include a causative hypothesis in our model (Figure 2; double‐headed arrow). The variance of each continuous variable was included as well (Figure 2; double‐headed circular arrows). Model fit was confirmed using a Chi square test of lack of fit test. To gain more clarity on the trade‐off relationships, we further investigated the effect of autotomy on each with individual LMMs.

#### 2.3.2 Analysis 2

To investigate the effect of increasing weapon loss on testes mass in a more detailed way, we built an LMM with testes mass as the response variable. Treatment was our categorical, independent variable, and body mass, as well as its interaction with treatment, were both included as fixed effects. Body mass was standardized to include the dry mass of the whole body, not including the mass of any remaining hind legs or the testes (Figure 1A + B), to avoid part‐whole correlation (Tomkins and Simmons 2002).

#### 2.3.3 Analysis 3

We further investigated the impact of increasing weapon loss on absolute flight muscle investment with flight muscle mass as the response variable. Treatment was our categorical, independent variable, and body mass a covariate. Body mass in Analysis 2 was calculated as the remaining dry mass of the body, not including any remaining hind legs, minus the mass of the testes (Figure 1A + C), again to avoid part‐whole correlation (Tomkins and Simmons 2002). Weapon mass was not included in this covariate to determine whether investment differed when body mass was similar across treatments. To account for possible size‐dependent effects of treatment on flight muscle mass, the interaction between treatment and body mass was included.

#### 2.3.4 Analysis 4

Losing one or both weapons itself changes eventual adult body mass, and therefore the mass that must be lifted off the ground when flying. We thus constructed a second LMM to determine how weapon loss changed the size of flight muscles relative to the mass lifted. In contrast to Analysis 3, here our goal was to determine whether autotomy caused a change in the ratio between the size of the flight muscle, and the mass needed to lift during flight; that is, flight muscle investment as relevant to the function of flight. The LMM of Analysis 4 was identical to that of Analysis 3, except that it included the mass of any remaining weapon in body mass (Figure 1A + C + D). We refer to this measure of body mass as “total lift mass” to specify that it includes the mass of remaining hind legs. Because the interaction with body size was not significant, it was dropped, and the model was rerun. We then extracted the estimated marginal means and compared them using a post hoc Tukey's test (Package: emmeans; Version: 1.8.9) (Lenth 2023).

## 3 Results

In Analysis 1, our structural equation model fit the data well (lack of fit test: 𝜒2 = 1.599, df = 1, *p* = 0.206). We found a positive correlation between our response variables—testes mass (coefficient = 0.763, *Z*‐value = 22.048, *p* < 0.0001) and flight muscle mass (coefficient = 0.850, *Z*‐value = 36.163, *p* < 0.0001)—and body mass (Figure 2). We did find a significant, positive correlation between autotomy status and testes mass; males that lost legs had larger testes (coefficient = 0.096, *Z*‐value = 5.000, *p* < 0.0001; Figure 2). Treatment was not found to have a statistically significant effect on flight muscle mass (coefficient = −0.013, *Z*‐value = 0.013, *p* = 0.345; Figure 2). Finally, when controlling for treatment and body size, a small but statistically significant positive correlation was found between testes and flight muscle (coefficient = 0.019, *Z*‐value = 7.004, *p* < 0.0001; Figure 2).

**FIGURE 3:**
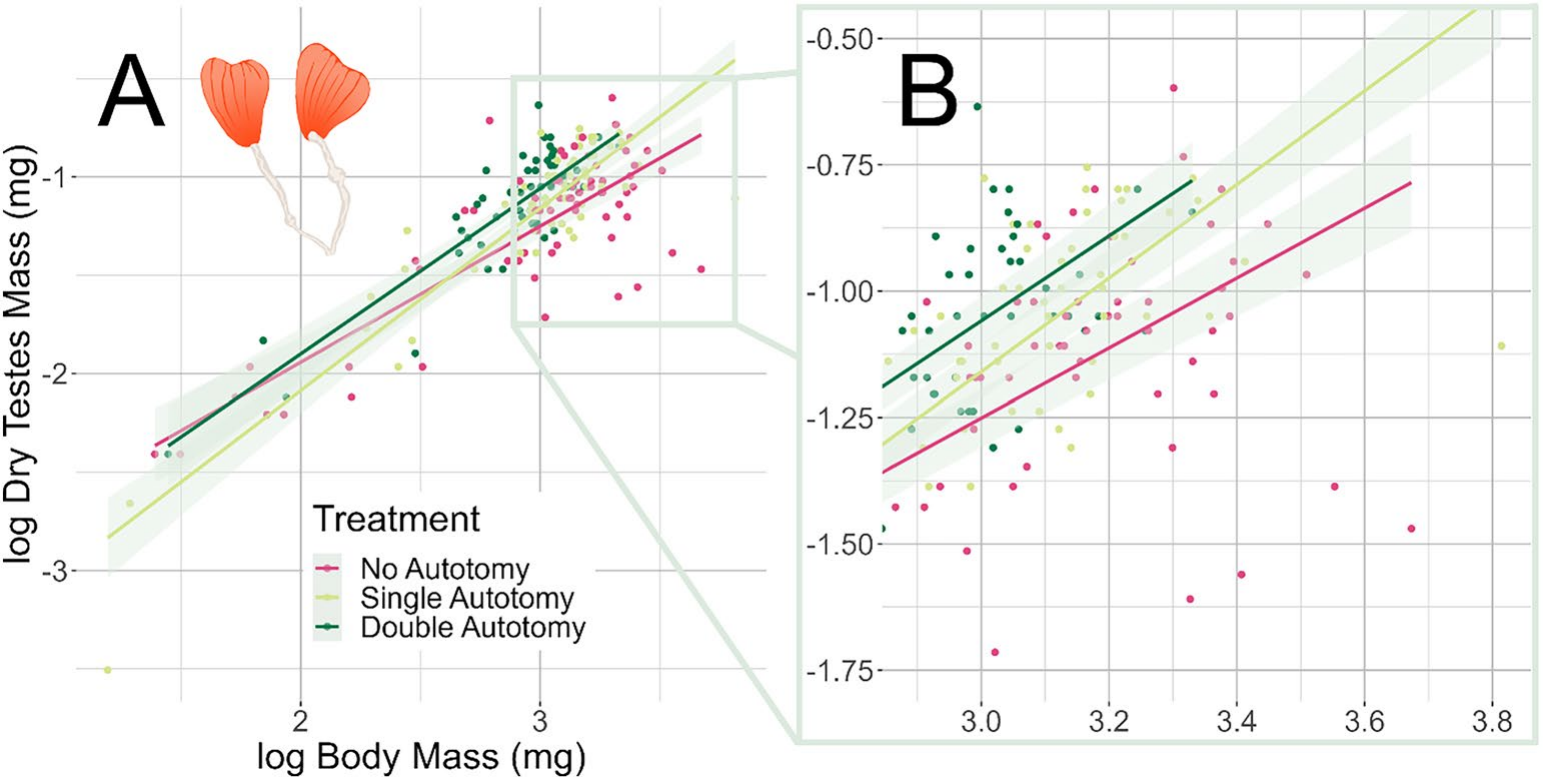
Males that lost one or both hind limbs grew larger testes than those that did not. Within autotomized males, those that lost both legs had larger testes than those that lost one. The full range of testes mass against body mass is shown in Panel A, with only the upper three quartiles by body mass (*n* = 153) highlighted in Panel B. The remaining lowest quartile of body mass (*n* = 51) did not appear to respond to autotomy by visual inspection. Quartiles are shown here for illustrative purposes only and were not used in statistical analyses. Shaded regions represent 95% confidence intervals. Illustration of the paired testes of *Narnia femorata* is shown in the upper left corner.

In Analysis 2, we dove deeper into the specific relationship between testes and weapons. We found a significant interaction between treatment and body size, indicating the effect of autotomy on testes mass was different across the range of male body sizes (Table 1). There were relatively few individuals at the smaller body sizes which appeared by visual inspection to be driving the interaction. The upper three quartiles of the data were at intermediate to large body sizes, where the size specific effect appeared negligible (Figure 3A). Looking at the majority of the data, males that lost both legs generally had larger testes than those that lost one, except among the very largest males were their testes are of a similar size (Table 1). Additionally, there was a strong relationship between weapon loss and testes mass: the greater the number of weapons lost, the greater the growth in testes (Figure 3B). This was similar to the result of our path analysis. We found that males that lost one hind leg had on average 9.2% greater testes mass than intact males, and males that lost both hind legs experienced an average increase of 21.1% in testes mass compared to those that lost no legs (Table 1 and Figure 3A).

**TABLE 1:** Wald chi‐square results from the linear mixed models. Interactions that were not statistically significant were dropped from the models before they were run again.

Analysis	*n*	Response	Factor	*χ* ^2^	df	*p*
2	186	Dry testes mass	Autotomy	32.781	2	< 0.0001*
			Body mass	575.786	1	< 0.0001*
			Autotomy × Body mass	13.414	2	0.0012*
3	186	Dry flight muscle mass	Autotomy	4.765	2	0.0923
			Body mass	1266.517	1	< 0.0001*
			Autotomy × Body mass	8.922	2	0.0116*
1	177	Dry flight muscle mass	Treatment	52.153	2	< 0.0001*
			Total lift mass	1254.441	1	< 0.0001*

* *p*‐value < 0.05.

**FIGURE 4:**
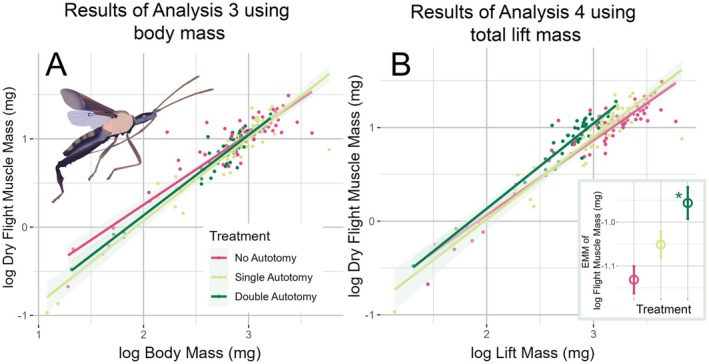
We looked at flight muscle in two ways. First, the results of Analysis 3 in Panel A show that flight muscle mass had little response to weapon loss during development—only the very smallest intact males had greater muscle mass than males that dropped a single hind‐leg weapon during development. In Panel A our analysis of body mass did not include the mass of any remaining weapons. In Analysis 4, with results shown in Panel B, we included the remaining weapon mass in body mass. We found that males that dropped both weapons during development had relatively greater flight muscle mass. Estimated marginal means (EMM), extracted from our model, are displayed with standard errors in the inset in the bottom right. The star indicates a statistically significant difference relative to other treatments. An illustration of flight muscle imposed over an illustration of a flying *Narnia femorata* is shown in the upper lefthand corner of Panel A.

In Analysis 3, we further investigated the impact of weapon loss on flight muscle, adjusted for body mass. Flight muscle mass was on average 14.5% of body mass, irrespective of treatment (NA: 14.97%; SA: 14.07%; DA: 14.53%), similar to the results of our path analysis. While there was no main effect of autotomy treatment on the mass of flight muscles (Table 1, Analysis 3), we did find a significant interaction between autotomy treatment and body size. Similarly to Analysis 2, this interaction only appeared to impact the smallest individuals by visual inspection. Intact males had larger flight muscles than males that lost a single leg. Males that lost both hind legs overlapped with both of these groups, however. Again, looking at the upper majority of the data by body mass, we found little trace of the interaction (Figure 4A).

Next, we took a functional view, as Analysis 3 did not take into account how much mass the animal would need to lift when flying. Weapons represent a substantial portion of body mass, composing an average 26.8% of body mass across males that did not receive autotomy. Losing weapons therefore reduced the weight an individual would need to lift in flight. In Analysis 3, we used the total mass of the insect, *including* the mass of any remaining leg weapons (here called total lift mass) as a covariate. The results from Analysis 4 revealed that males that lost both weapons had heavier flight muscles relative to total mass lifted when compared to males that retained one or both hind legs (NA–DA: estimate = −0.1748, df = 169, *p* = < 0.0001; SA–DA: estimate = −0.1385, df = 163, *p* = < 0.0001; NA–SA: estimate = −0.0363, df = 168, *p* = 0.296; Table 1; Figure 4B). Flight muscles accounted for a smaller percentage of total lift mass in males that still possessed both weapons (NA: 11.79%), compared to those that lost one (SA: 12.39%), or especially both weapons (DA: 14.53%). Males lacking weapons entirely therefore had less mass to maneuver than males retaining weapons, but had similarly sized flight muscles to retaining weapons.

Curiously, eight males (3.7%) were noted during the study to be highly pigmented with exceedingly soft cuticles. These males were all on the low end of body mass and were kept in the analysis because it was not clear what caused this phenotype to emerge. They were diverse in both treatment and parentage. Finally, we found that pronotum width decreased very slightly with autotomy (Figure S1); however, this difference in body size was not detectable in our other proxy, body mass (Table S1).

## 4 Discussion

Here, we examined investment dynamics in flight muscle, a sexually selected weapon, and testes. Leaf‐footed bugs provided us an experimental tool to investigate this relationship, autotomy, which we capitalized on by inducing the loss of one or two weaponized hind legs during development. In our path analysis, we found that autotomy does indeed increase absolute investment in testes, but does not increase direct investment in flight muscle tissue. In our more detailed analysis, we found a stepwise increase in testes mass, as predicted. Males that lost one hind leg had an average of 9.2% greater testes mass than intact males, and males that lost both hind legs experienced an average increase of 21.1% in testes mass compared to those that lost a single leg. Our individual model on flight muscle did not yield general evidence of a trade‐off between hind‐leg growth and flight muscle. It is worth noting; however, that males that dropped both hind‐leg weapons had substantially reduced lift mass. Thus, the demands on their flight muscles should be greatly reduced, facilitating dispersal. These males may thus be able to move around with greater ease and find females not guarded by other males. More explicit tests of dispersal in the environment, and the impacts of leg weight on flight mechanics are needed to confirm this.

Previous work has shown that male *Narnia femorata* that drop one leg during development grow larger testes (Joseph et al. 2018; Miller et al. 2019, 2021; Greenway et al. 2023). Further, the increase in testes mass found in *N. femorata* has been linked to an increase in sperm production (Cavender et al. 2021), greater duration of mating (Greenway et al. 2023), and increased fitness in non‐competitive contexts (Cirino et al. 2021). Somjee, Miller, et al. (2018) and Somjee, Woods, et al. (2018) found that male *Mictis profana* grow larger testes with the loss of two legs, but this work did not include a single leg autotomy manipulation. Here, in a single study, we found that males that lost two hind‐leg weapons during development grew testes with approximately two times the increase in mass relative to those that lost just one hind‐leg weapon. This fascinating increase suggests that the loss of a hind limb is not only a cue for increased testes growth, but that the resources invested in weapons come at a direct cost to testes. This pattern is relevant because autotomy is common in leaf‐footed bugs (Emberts et al. 2016) and it leads to reduced success in male–male competition in *N. femorata* (Emberts et al. 2018). Thus, leg loss could serve as a cue for reduced mating opportunities and heightened sperm competition. In that case, simply responding to the cue by growing larger testes may allow males to compensate for their lack of competitive prowess. But, here, these results further support evidence that leg loss may be serving as more than a cue, and that a direct resource allocation trade‐off is at play. Greenway et al. (2023) found a strong positive association between testes growth and the quantity of “savings” an individual reaped from not growing a hind leg. Testes and hind leg weapons develop at the same time, during the final two instars of juvenile development, and it appears that the use of resources for legs drains those resources available for testes growth. This is consistent with findings in other insect systems, where manipulations to inhibit investment in weapons lead to a concurrent investment in testes mass (Simmons and Emlen 2006; Simmons et al. 2017), and inhibition of genitals led to increased investment in weapons (Moczek and Nijhout 2004). Taken together, the experimental work on insects supports an underlying energetic relationship between postcopulatory investment and weapons.

Although the general findings are clear, Figure 3 suggests that the smallest males did not grow larger testes in response to autotomy. Such a pattern might be expected because small, poor‐condition individuals may have to prioritize survival over reproduction (Stearns 1998), at least shortly after sexual maturity when these animals were frozen. These males may have redirected the hind‐leg investment elsewhere, just not to their testes. Insects may be able to increase testes size later on in adulthood, and so these young males may not yet have prioritized testes growth.

We found, when considering absolute investment only, the mean flight muscle mass of most body sizes was mostly invariant across the three treatments. In our individual analysis 3, there was a slight decrease in flight muscle mass among the smallest males that lost one leg compared to those that retained both their legs. Males missing one hind leg overlapped with both of these groups. The biological relevance of this trend among these small males is not clear, and the majority of males showed no change in flight muscle due to autotomy. More explicit investigations of the smallest individuals are needed to better understand this trend. Among the majority of males; however, invariance in flight muscle investment may be due to strong selection to retain flight capacity, as there is a minimum flight muscle to mass ratio insects must reach to attain flight (Marden 2000).

When considering the total mass a male would need to lift to get off the ground, we found that males that lost both hind leg weapons had heavier flight muscles. This result is consistent with negative relationships between dispersal and reproduction found in other insects (Heinze and Hölldobler 1993; Saglam et al. 2008; Chang et al. 2021); however, the results from our second analysis imply that the pattern here does not result from a straightforward shift in investment. As the hind leg weapons of adult leaf‐footed bugs are enlarged and often heavy, males that dropped hind‐leg weapons during development grew into lighter adults. Thus, instead of decreased reproductive investment causing an increase in flight muscle growth, weaponless males were freed from the cost of carrying weapons (26.8% of body mass in males retaining both legs), resulting in a greater proportion of overall mass contributed to flight muscle. The proportion of mass contributed to flight muscle is typically positively associated with flight performance (Marden 1987, 2000; Fischer and Kutsch 2000), and work in other insects has found sexually selected traits to pose a substantial cost to locomotion (Madewell and Moczek 2006; Allen and Levinton 2007; Goyens, Dirckx, and Aerts 2015), including flight (Ribak and Swallow 2007; Goyens, Van Wassenbergh, et al. 2015; Menezes and Palaoro 2022; but see McCullough and Tobalske 2013). Thus, we expect lighter‐weight insects that maintain a high investment in flight muscle might be able to achieve longer or more frequent flights. Further, as males entirely lacking weapons are likely to be at a significant disadvantage when competing against males with even one weaponized leg (Emberts et al. 2018), the increase in total mass contributed to flight muscle could help them seek out new territories and mates (Fujisaki 1981; Miller 2007). These males may even have an advantage in finding potential mates without nearby rivals (Parker et al. 2013). Our findings present an exciting vein of future inquiry investigating the dynamics of flight in doubly autotomized *N. femorata* to explore the utility of missing weapons to flight and dispersal in the wild.

The evidence of a potential adaptive shift in investment strategy was also found in our path analysis, where we found a positive correlation between flight muscle and testes mass. That is, controlling for autotomy treatment and body size, males that invested more in their flight muscles also invested more in their testes. Males with greater sperm competitive capacity appear to have a greater capacity to disperse, which may enable them to fly to many cactus plants, thus maximizing their mating success. Responses to weapon loss therefore could represent discrete strategies, similar to alternative mating strategies (Shuster and Wade 2003; Shuster et al. 2019). Alternatively, the positive correlation we found in our path analysis could be the result of greater acquisition of energy in large males supporting greater investment in all traits (van Noordwijk and de Jong 1986; Reznick et al. 2000; Supriya et al. 2018). Such positive relationships are common, and thus explicit manipulations of resource acquisition or dispersal traits are needed to clarify causation here.

The males in the lowest quartile of body mass in the current study showed markedly different trends from the majority of males. They lacked a discernible response in testes mass to autotomy, and had only a slight, mass‐specific response in flight muscle investment. Previous studies have not found that males of low body size respond differently to manipulations (Emberts et al. 2017; Joseph et al. 2018; Somjee, Miller, et al. 2018; Miller et al. 2019, 2021), and it is not obvious what was so different about these males apart from their size. As insects were maintained on live plants, natural variation in nutrition may be responsible for these small bugs; however, individuals in depauperate resource environments are expected to show more exaggerated trade‐offs, not less exaggerated as observed here (van Noordwijk and de Jong 1986; Reznick et al. 2000; Supriya et al. 2018). Given this apparent contradiction, future work should focus on the smallest males in an effort to better understand investment dynamics at the extremes of available resources.

Our study demonstrates the value of manipulative experiments on resource‐allocation trade‐offs that measure multiple traits. Flight muscle appeared somewhat canalized, or unchanged, in response to a changing environment, possibly due to evolutionary constraints maintaining flight muscle mass at a size necessary to attain flight (Marden 2000). As a result of this canalization, males that lose weapons may be at an advantage when flying long distances, as they weigh much less than males retaining hind legs. The rigidity of flight muscle mass is contrasted by the flexibility in testes investment, which increases as weapons are lost during development. Individual trait responses to changes in investment strategy are likely diverse and may vary across populations or species (Garland et al. 2022). Our results show that canalized traits may be deeply involved in trade‐offs, either as unavoidable sinks of energy or as potentially exploitable new strategies. We found a positive correlation between flight muscle mass and testes mass, further supporting the idea that autotomy may cause an overall shift in reproductive strategy, not just between two traits. Selection's action on such investment strategies may represent early stages of alternative reproductive strategies and provide purchase for selective change. Future work should investigate the fitness implications of these investment decisions to better predict the action of selection.

## Author Contributions

**James C. Boothroyd:** conceptualization (equal), data curation (equal), formal analysis (equal), methodology (equal), project administration (equal), visualization (lead), writing – original draft (lead), writing – review and editing (lead). **Steve M. Smit:** conceptualization (equal), data curation (equal), investigation (equal), methodology (equal), project administration (equal), writing – review and editing (supporting). **Niko S. Zlotnik:** conceptualization (equal), data curation (equal), investigation (equal), methodology (equal), project administration (equal), writing – review and editing (supporting). **Christine W. Miller:** conceptualization (equal), formal analysis (equal), funding acquisition (lead), methodology (equal), project administration (equal), resources (lead), supervision (lead), writing – original draft (equal), writing – review and editing (lead).

## Conflicts of Interest

The authors declare no conflicts of interest.

## Supporting information


Data S1

## Data Availability

All code and data are accessible via Dryad: https://doi.org/10.5061/dryad.k0p2ngfkz.
