# Supplementary material for: Loss of Paired Weapons Leads to Larger Testes and a Lighter Load for Dispersal
Source: Ecol Evol. 2025 Jul 6;15(7):e71724. doi: 10.1002/ece3.71724 (PMC12229949; doi:10.1002/ece3.71724)
Supplement: Supplementary file 1 — Data S1 [file ECE3-15-e71724-s001.docx]

**This file includes:**

**Text S1:** Supplementary Methods

**Text S2:** Supplementary Discussion

**Table S1:** Results of the generalized linear mixed models of body size

**Figure S1:** Graph showing estimated marginal means of a linear measure of body size between treatments

## Text S1: Supplementary Methods

Voluntary limb loss can be associated with myriad costs [1, 2], which, if incurred during development, may stunt growth and reduce adult body size [3]. Therefore, to determine whether autotomy during development imposed substantial costs on *Narnia femorata*, we investigated the effect of losing one or both hind limbs on pronotum width. Pronotum width is a good linear proxy for body size in this species [4], and is fixed after adult eclosion [3].

Photographs were taken of each male prior to dissection using a Canon EOS 50D digital camera. Image J software v1.46r [5] was used to measure pronotum width. We tested for effects of treatment on pronotum width and body mass using linear mixed models (LMM) (Package: lme4; Version: 1.1.35.1) [6], where autotomy status was a categorical explanatory variable, and family was included as a random effect. We then extracted the estimated marginal means and compared them using a post-hoc Tukey’s test (Package: emmeans; Version: 1.6.1).

We used a Wald Chi square test to detect statistical significance in our model (Package: rstatix; Version: 0.7.2) [7]. All statistical analyses were performed using R version 4.3.2 (2023-10-31 ucrt) [8], and all variables were log transformed prior to analysis.

## Text S2: Supplementary Results and Discussion

Males that lost hind legs during development grew to be 3% smaller adults on average, than those that retained their legs (GLMM: Wald χ2=6.7294, p=0.03457; Table S1). (Figure S1). Interestingly, the small change in body size did not translate into a difference in total body mass (GLMM: Wald χ2=1.1936, p=0.5506; Table S1).

In our study, contrary to previous work [9-13], males that lost hind limbs at all grew about 3% smaller than those that retained both their limbs. This result could mean there is some cost to limb loss during development which does not scale with the number of limbs lost. Despite the apparently minimal trauma caused by autotomy, it always leaves a small break in the haemocoel, against which animals may mount immune responses [14]. Additionally, we did not detect a difference in total body mass, which may fluctuate throughout an insect’s adult life. Pronotum width, by contrast, captures body size fixed at adulthood. Males may therefore have been able to recoup some losses in the two weeks between adult eclosion and when they were frozen. This could explain why autotomy resulted in slightly smaller linear measure of body size, which was not detectable in body mass.

The trend of reduced body size is not apparent in previous studies; however, there were two key differences between the insects here, and those in prior studies. First, insects in the present study were the offspring of two different populations of *N. femorata*, and thus were genetically distinct from the insects used in other studies. Second, previous insects were reared in a greenhouse environment, where temperatures were likely much more variable than the controlled, indoor environments the present insects were reared in. Temperature has a significant impact on growth rate and development time in ectotherms [15], and the reduced thermal variability may have allowed previously obscured trends in body size to become more apparent. Given the small effect size on our linear measure of body size, it seems that any impact autotomy has on body size is small, and is likely not present to a measurable degree in wild populations, where conditions are much more variable that in rearing rooms or greenhouses.

**Table S1** Results of the generalized linear mixed models of body size.

| n | Response | Factor | χ2 | Df | p |
| --- | --- | --- | --- | --- | --- |
| 206 | Pronotum Width | Autotomy | 6.7294 | 2 | 0.03457* |
| 186 | Total Body Mass | Autotomy | 1.1936 | 2 | 0.5506 |


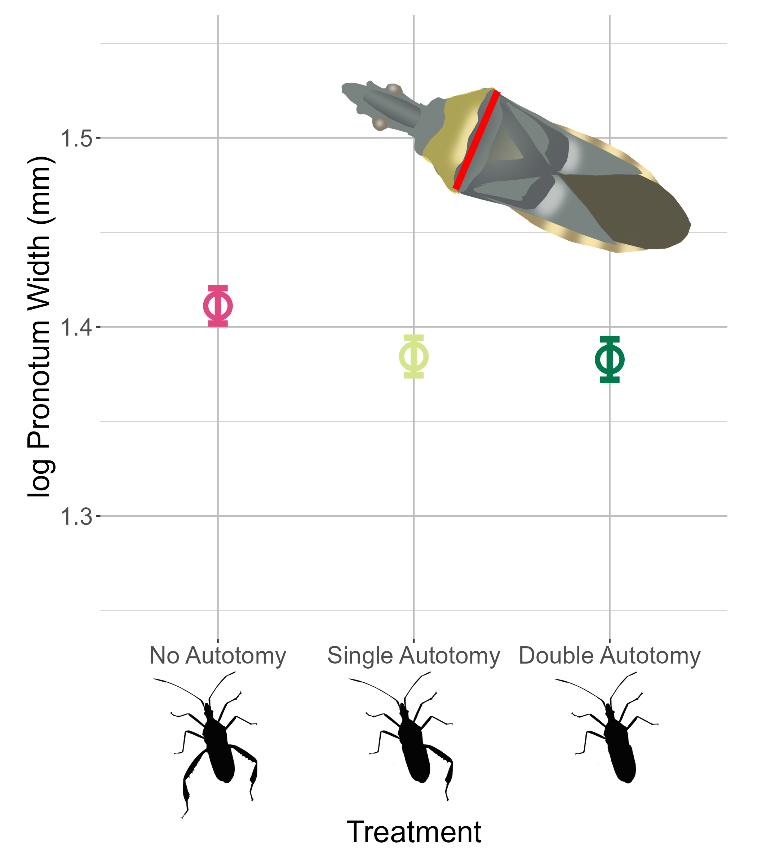


**Figure S1.** Males that dropped one or two hind limbs during development grew a slightly smaller adult body (measured as pronotum width). Estimated marginal means are shown with standard errors. An illustration of a *N. femorata* male is shown in the upper right, with a red line drawn to indicate pronotum width.

## References

1. Emberts, Z, Escalante, I & Bateman, PW. 2019 The ecology and evolution of autotomy. *Biological Reviews* **94**, 1881-1896.

2. Maginnis, TL. 2006 The costs of autotomy and regeneration in animals: a review and framework for future research. *Behav Ecol* **17**, 857-872.

3. Nijhout, H. 2003 The control of body size in insects. *Dev Biol* **261**, 1-9.

4. Gillespie, SR, Tudor, MS, Moore, AJ & Miller, CW. 2014 Sexual selection is influenced by both developmental and adult environments. *Evolution* **68**, 3421-3432. (doi:10.1111/evo.12526).

5. Schneider, CA, Rasband, WS & Eliceiri, KW. 2012 NIH Image to ImageJ: 25 years of image analysis. *Nature Methods* **9**, 671-675. (doi:10.1038/nmeth.2089).

6. Bates, D, Mächler, M, Bolker, BM & Walker, SC. 2015 Fitting Linear Mixed-Effects Models Using lme4. *Journal of Statistical Software* **67**, 1-48. (doi:10.18637/jss.v067.i01).

7. Kassambara, A. 2023 rstatix: Pipe-Friendly Framework for Basic Statistical Tests. (

8. Team, RC. 2021 R: A Language and Environment for Statistical Computing. (R Foundation for Statistical Computing.

9. Emberts, Z, Miller, CW, Kiehl, D & St Mary, CM. 2017 Cut your losses: self-amputation of injured limbs increases survival. *Behav Ecol* **28**, 1047-1054. (doi:10.1093/beheco/arx063).

10. Joseph, PN, Emberts, Z, Sasson, DA & Miller, CW. 2018 Males that drop a sexually selected weapon grow larger testes. *Evolution* **72**, 113-122. (doi:10.1111/evo.13387).

11. Miller, CW, Joseph, PN, Kilner, RM & Emberts, Z. 2019 A weapons-testes trade-off in males is amplified in female traits. *Proc. R. Soc. B* **286**. (doi:10.1098/rspb.2019.0906).

12. Miller, CW, Joseph, PN & Emberts, Z. 2021 Trade-offs between weapons and testes do not manifest at high social densities. *J Evol Biol* **34**, 726-735. (doi:10.1111/jeb.13790).

13. Somjee, U, Miller, CW, Tatarnic, NJ & Simmons, LW. 2018 Experimental manipulation reveals a trade-off between weapons and testes. *J Evol Biol* **31**, 57-65. (doi:10.1111/jeb.13193).

14. Ardia, DR, Gantz, JE, Schneider, BC & Strebel, S. 2012 Costs of immunity in insects: an induced immune response increases metabolic rate and decreases antimicrobial activity. *Funct Ecol* **26**, 732-739. (doi:10.1111/j.1365-2435.2012.01989.x).

15. Chakraborty, A, Walter, GM, Monro, K, Alves, AN, Mirth, CK & Sgro, CM. 2023 Within-population variation in body size plasticity in response to combined nutritional and thermal stress is partially independent from variation in development time. *J Evol Biol* **36**, 264-279. (doi:10.1111/jeb.14099).
